# The dynamic feasibility of resisting (R), accepting (A), or directing (D) ecological change

**DOI:** 10.1111/cobi.14331

**Published:** 2024-07-17

**Authors:** Amanda E. Cravens, Katherine R. Clifford, Corrine Knapp, William R. Travis

**Affiliations:** ^1^ Forest and Rangeland Ecosystem Science Center U.S. Geological Survey Corvallis Oregon USA; ^2^ Western Water Assessment University of Colorado Boulder Boulder Colorado USA; ^3^ Haub School of Environment & Natural Resources University of Wyoming Laramie Wyoming USA; ^4^ Department of Geography and North Central Climate Adaptation Science Center, Cooperative Institute for Research in Environmental Science University of Colorado Boulder Boulder Colorado USA

**Keywords:** climate adaptation, ecological transformation, financial constraints, organizational culture, public opinion, regulations, resist–accept–direct framework, social–ecological feedbacks, adaptación climática, cultura de organización, marco resistir‐aceptar‐dirigir, opinión pública, regulaciones, retroalimentación socio‐ecológica, restricciones económicas, transformación ecológica

## Abstract

Ecological transformations are occurring as a result of climate change, challenging traditional approaches to land management decision‐making. The resist–accept–direct (RAD) framework helps managers consider how to respond to this challenge. We examined how the feasibility of the choices to resist, accept, and direct shifts in complex and dynamic ways through time. We considered 4 distinct types of social feasibility: regulatory, financial, public, and organizational. Our commentary is grounded in literature review and the examples that exist but necessarily has speculative elements because empirical evidence on this newly emerging management strategy is scarce. We expect that resist strategies will become less feasible over time as managers encounter situations where resisting is ecologically, by regulation, financially, or publicly not feasible. Similarly, we expect that as regulatory frameworks increasingly permit their use, if costs decrease, and if the public accepts them, managers will increasingly view accept and direct strategies as more viable options than they do at present. Exploring multiple types of feasibility over time allows consideration of both social and ecological trajectories of change in tandem. Our theorizing suggested that deepening the time horizon of decision‐making allows one to think carefully about when one should adopt different approaches and how to combine them over time.

## Abstract (es)

La viabilidad dinámica de resistir (R), aceptar (A) o dirigir (D) el cambio ecológico

**Resumen:**

Las transformaciones ecológicas ocurren por el cambio climático, lo que representa un reto para los enfoques tradicionales para decidir en torno a la gestión de tierras. El marco resistir‐aceptar‐dirigir (RAD) ayuda a los gestores a considerar cómo responder a este reto. Analizamos cómo la viabilidad de las opciones para resistir, aceptar y dirigir cambia de manera compleja y dinámica con el tiempo. Consideramos cuatro tipos distintos de viabilidad: regulatoria, económica, pública y de organización. Nuestro comentario está basado en la revisión bibliográfica y los ejemplos que existen, pero por necesidad tiene elementos especulativos ya que la evidencia empírica sobre esta estrategia emergente de gestión es escasa. Esperamos que las estrategias de resistir se vuelvan menos viables con el tiempo conforme los gestores encuentren situaciones en las que resistir no es viable de forma ecológica, económica, pública o por regulación. Al igual esperamos que cada vez más los marcos regulatorios permitan su uso, si el costo disminuye, y si el público los acepta, los gestores verán cada vez más viables las estrategias de aceptar y dirigir que las que utilizan actualmente. La exploración de varios tipos de viabilidad a lo largo del tiempo permite considerar las trayectorias sociales y ecológicas del cambio en conjunto. Nuestra teoría sugiere que profundizar en el horizonte temporal de las decisiones permite que se analice con cuidado sobre cuando se deben adoptar enfoques diferentes y cómo combinarlos con el tiempo.

## INTRODUCTION

Responding strategically to strong, human‐driven environmental change is a profound and growing challenge for natural resource managers (Schuurman et al., 2022, 2020). The combination of climate change and a range of anthropogenic stressors, including land use change, pollution, and introduction of non‐native species (e.g., Bates et al., 2017; Christensen et al., 2006; Jenny et al., 2020), is resulting in ecological transformation, defined by Crausbay et al. (2022, p. 72) as “the dramatic and irreversible shift in multiple ecological characteristics of an ecosystem, the basis of which is a high degree of turnover in ecological communities.” Inherent in the challenge of ecological transformation is the “nonstationarity” of future climate conditions (Milly et al., 2008), which often creates disparities between emerging ecological realities and ecological objectives that were set based on historical baselines (Biggs et al., 2018; Millar et al., 2007; Schuurman et al., 2022; West et al., 2009). As Crausbay et al. (2022, p. 71) point out, “the rates and magnitudes of modern global change challenge the viability of longstanding management philosophies, cultures, and mandates built on the assumption that the climate of the future—and therefore what is ecologically possible in a given place—will reflect the past (e.g., US Forest Service's 2012 Forest Planning Rule, US Fish and Wildlife Service policies on habitat management in wildlife refuges).”

In response to this challenge, the resist–accept–direct (RAD) framework has emerged to aid managers’ decisions about how to respond to new ecological trajectories in a nonstationary world (Lynch et al., 2021; Schuurman et al., 2022, 2020; Thompson et al., 2021). The RAD framework offers 3 broad categories of management actions. Managers can use resist strategies to steer an ecosystem back toward previous conditions; the accept strategy to not intervene or attempt to steer an ecosystem toward a specific ecological outcome; or direct strategies in the hope of guiding an ecosystem to a new condition (Table 1). There is not a universal best choice (Clifford et al., 2020); rather, the goal is to ask managers to consider all 3 options and then determine which is best for their context (Magness et al., 2022). In other words, the framework is meant to aid managers in considering the full suite of alternatives rather than maintaining their default approach (e.g., resisting because they have always managed for historical conditions).

**TABLE 1:** Comparison of resist (R), accept (A), and direct (D) approaches to responding to ecological transformation in terms of what each involves, underlying goals and values, and possible motivations for choosing an approach.

Category	Resist change	Accept change	Direct change
How is the approach defined?	Work to maintain or restore ecosystem composition, structure, or function on the basis of historical or acceptable current conditions.	Allow ecosystem composition, structure, and function to drift autonomously (away from historical conditions) without intervening to alter the ecological trajectory.	Actively shape ecosystem composition, structure, and function to create a new ecosystem configuration on the basis of preferred conditions and ecosystem services.
Desired outcome or goals	Historical conditions and services persist or are restored based on a retrospective benchmark.	New conditions and services result from intentionally not guiding change.	New conditions, clearly defined, are intentionally sought and ideally part of a self‐sustaining system.
		No specific target conditions are needed.	
		Allocation of finite management resources to other focal areas or issues are strategic.	
Motivations for each approach	Historical or current conditions are conserved.	Some ecosystems in an unmanipulated condition are conserved.	A new set of conditions and ecosystem services are provided that are preferable to those that would result from either accepting change or seeking to resist change where doing so is futile.
	Existing ecosystem services are retained or former ecosystem services are recreated.	Resources or inability to shape the ecological trajectory are insufficient.	
	Buy time for autonomous species response or further management actions.	Desirable ecosystem services are not threatened.	

*Note*: Adapted from Schuurman et al. (2022, 2020).

**FIGURE 1:**
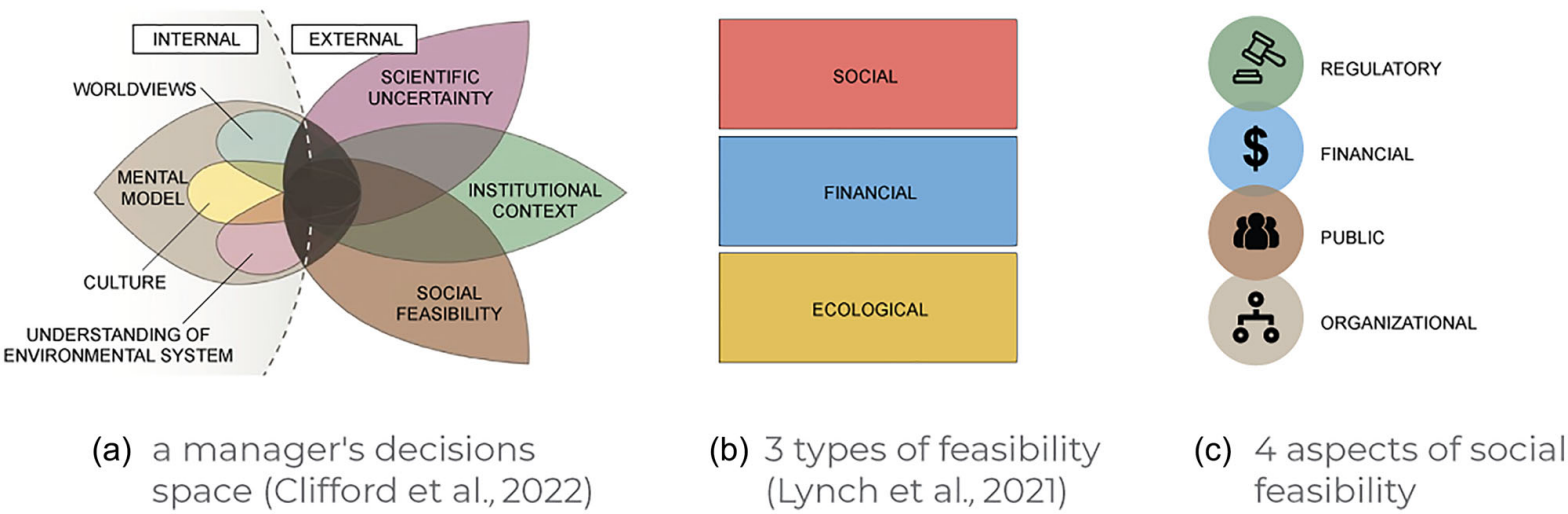
Following (a) Clifford et al.’s (2022) model of a manager's decision space when responding to ecological transformation, (b) Lynch et al.’s (2021) social feasibility is divided into 3 additional types to yield (c) 4 aspects of feasibility that shape future decisions to resist, accept, or direct change.

Despite the widespread interest in and growing use of the RAD framework, particularly by federal agencies in the United States (e.g., National Park Service, 2021a; U.S. Department of Interior, 2021; U.S. Fish & Wildlife Service, 2022), determining which strategies to implement in a specific place in response to a given ecological trajectory is anything but straightforward. The description of constraints on decisions in Clifford et al. (2022) and of feasibility in Lynch et al. (2021) provides an important starting point to understand the diverse factors that influence the feasibility of RAD decisions (Figure 1a,b). However, these papers primarily address decision‐making at one moment in time. This formulation mirrors the structure of management plans, which often take years to gestate and are aimed at defining a set of actions to reach a preferred resource condition (e.g., Christensen et al., 1996; NPS, 2021a, 2021b). However, using RAD in a changing socioecological context implies making successive RAD decisions over time.

To address this more realistic scenario of making multiple decisions about ecological transformation over time, one must account for the complex reality that the social system possesses multiple dynamic trajectories that shift as humans respond and adapt to ecological changes. Although detailed attention has focused on ecological change over time, to date work on ecological transformation and the RAD framework has generally treated social systems as stationary, neglecting the reality that social and ecological systems both independently change and also influence one another. In the parlance of socioecological systems theory (Pulver et al., 2018), researchers and managers thus far have insufficiently accounted for feedbacks between the social and ecological systems (Blythe et al., 2017; Larrosa et al., 2016).

We argue that the factors that shape decisions to resist, accept, or direct evolve over time. A future RAD decision is thus shaped not only by the future ecological trajectory, but also by the dynamic trajectories of multiple human drivers and constraints. We combined Lynch et al.’s (2021) dimensions of feasibility and Clifford et al.’s (2022) visualization of a manager's decision space to describe the multiple, intersecting aspects of feasibility that shape successive RAD decisions. Feasibility represents, at the most basic, an evaluation of viability or practicality (Ulibarri et al., 2021). It has been defined as “the possibility that [something] can be made, done, or achieved, or is reasonable” (Cambridge Dictionary https://dictionary.cambridge.org/dictionary/english/feasibility). In mathematical approaches to decision analysis, feasibility is conceived as the set of possible decisions or outcomes that meet a given set of constraint criteria as specified by equations (Weirich, 2001). Although this approach is conceptual rather than mathematical, Clifford et al. (2022) reflected the intent of this view of feasibility in their conceptual framework for RAD decision‐making, which defined the decision space of a manager responding to a transforming ecological trajectory as the intersection of their (internal) mental model and external constraints, including institutions, stakeholders’ views, and the state of scientific knowledge (Figure 1a). Lynch et al. (2021) conceptualized feasibility as having 3 distinct dimensions: ecological, social, and financial (Figure 1b).

Managers will be confronted with a series of decision points as the ecological trajectory unfolds (Schuurman et al., 2022). In our conception, time and extent of ecological change are roughly correlated as one considers the feasibility of future decisions. (We did not consider the case of abrupt ecological or social change [Smith et al., 2022; Williams et al., 2021].) Inspired by the Clifford et al. (2022) model, we divided what Lynch et al. (2021) grouped into social feasibility into 3 distinct categories: regulatory feasibility (i.e., extent to which laws, policies, and administrative guidance permit or incentivize a choice [Hamlin, 2017]), public feasibility (i.e., the way that managers understand stakeholder and public attitudes as a perceived or actual constraint on decision‐making), and organizational feasibility (i.e., managers’ individual or collective understanding of a strategy as possible or desirable). We also include financial feasibility (Figure 1c). We asked what determines how each of these evolve over time and how might these changes influence the overall viability of each category of RAD strategy at a given decision point? Focusing primarily on the United States (but with occasional examples from farther afield), we moved from formal institutional structures (regulatory feasibility) to resource availability (financial feasibility) to constraints imposed by the views of stakeholders (public feasibility) and managers themselves (organizational feasibility).

Our analysis is necessarily speculative and based on logical extrapolation from our collective knowledge of a wide variety of social science theory and literature. This was unavoidable because implementation of RAD in the real world is still in its infancy, and predicting the future is inherently complex (Stirling, 2010). As such, our conclusions (and particularly the possible pathways as we have drawn them) should not be seen as predictive; rather, they should be taken as one set of possibilities for how these variables could interact and one set of pathways for how the future might unfold. Nonetheless, we sought to demonstrate why evaluating feasibility of RAD strategies requires considering social and ecological trajectories in tandem and paying attention to feedbacks between them.

## REGULATORY FEASIBILITY AND THE RULES THAT DETERMINE WHICH DECISIONS MANAGERS CAN MAKE

Regulatory feasibility is the extent to which laws, policies, and administrative guidance permit or incentivize a choice (Hamlin, 2017). Most often, climate adaptation scholars focus on regulations and laws as constraining innovative strategies (e.g., Gupta et al., 2010; Ulibarri et al., 2020), but policies and laws simultaneously reflect and shape the values and the preferences of society (Mahoney & Thelen, 2009). As a result, regulations are not fixed; rather, they change—gradually or abruptly—over time in response to changing conservation values and knowledge as well as larger societal forces (Coloff et al., 2017; Fidelman et al., 2019; Michelotta et al., 2017). At the same time, changes in laws or policies can shape agency decision‐making and ecological outcomes (e.g., Swette & Lambin, 2021). Scholars working on “adaptive governance” have even proposed proactively designing laws and policies to intentionally shift societal attitudes and behaviors in desirable directions to support adaptation to environmental change (DeCaro et al., 2017).

Regulations operate at a range of scales, and some elements are more likely to shift than others. Laws are generally most durable, especially in the case of long‐standing or foundational laws, such as the US National Park Service Organic Act (Public Law 64–235) or the Treaty of Waitangi governing relationships between the New Zealand government and Maori iwi, which has now been written into a range of New Zealand environmental and other statues by reference (NZ Ministry for Culture & Heritage, 2017). Administrative guidance on the interpretation of laws is often more malleable than statues themselves because the mechanisms that enable change are easier to achieve. For instance, the past 3 US presidential administrations have released guidance for implementing the National Environmental Policy Act (NEPA, Public Law 91−190; see CEQ, 2023; https://obamawhitehouse.archives.gov/administration/eop/ceq/initiatives/nepa; https://trumpwhitehouse.archives.gov/ceq/nepa‐modernization). This suggests that provisions incentivizing or requiring particular RAD strategies or conversely removing barriers to RAD strategies are likely to first appear in administrative guidance. For example, agencies might produce guidance detailing how future climate projections are to be considered in decision‐making, which might sway which strategies seem feasible to managers (e.g., California Ocean Protection Council, 2018).

Regulatory feasibility will shape each RAD strategy differently over time. Resist strategies generally have few regulatory barriers in the short term because they align well with traditional management paradigms that regulations were designed to address. However, as ecological trajectories progress and resisting becomes less ecologically feasible, regulations requiring specific outcomes may become constraining. For instance, the U.S. Endangered Species Act (ESA) (Public Law 93–205) requires maintaining populations of and habitat for threatened and endangered species. In some cases, such as that of Hawaiian honeycreepers (subfamily Carduelinae) (Fortini et al., 2020; Judge et al., 2021), managers are already facing situations where accomplishing what the ESA requires is extremely challenging or not possible with resist strategies.

**FIGURE 2:**
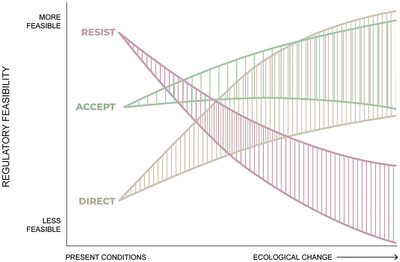
How regulatory feasibility of resist, accept, and direct strategies might evolve over time.

As society grapples with and responds to ecological transformation, we expect that accept and direct strategies will become more feasible (Figure 2), although direct options start with a decided regulatory disadvantage (Lieberman, 2017, 2018; Stephenson & Millar, 2012). We expect that resist strategies will become less feasible as laws and policies requiring specific outcomes become increasingly challenged by continuing ecological change. In Figure 2, curves represent one set of possible future trajectories and account for uncertainty about more distant futures.

At present, accept tends to be a strategy of exhaustion or last resort because current regulatory frameworks often require explicit management objectives (National Park Service, 2021b; West et al., 2009) (also see 54 U.S.C. ch. 1005 § 100502 and 16 U.S. Code § 1600 et seq) that serve as a barrier to intentionally choosing an accept strategy, except perhaps in wilderness areas, where doing nothing is viewed as consistent with the mandate to leave these lands “untrammeled” (Public Law 88‐577). Allowing greater space for choosing accept strategies may require greater guidance for determining management effectiveness in the lack of explicitly defined objectives. In the near term, direct strategies appear to be the least feasible approach from a regulatory standpoint. They represent the epitome of the paradigm shift away from using historical benchmarks to guide management objectives (Schuurman et al., 2022), and discussions are ongoing about the extent to which different forms of direct strategies are allowed under current law and policy (including when pilot projects and experimentation are or are not allowed [e.g., whether beaver dam analogs are permitted under current water law] [Pfaeffle et al., 2022]) (Lieberman, 2017, 2018; Stephenson & Millar, 2012). Additionally, regulators and governments tend to be risk adverse (Ulibarri et al., 2017), which means regulation can serve as a barrier to new management approaches until they are tested in pilot contexts or by the private sector (e.g., beneficial reuse of sediment to support coastal resilience [Ulibarri et al., 2020]). Similar to accept strategies, as ecological change trajectories proceed and as field‐level managers increasingly grapple with what is or is not permitted under which circumstances, agencies will likely develop more explicit guidance. For instance, the US Department of Interior is currently developing frameworks to aid its wildlife managers in determining if, when, and how to implement managed relocation or conservation introduction (Karasov‐Olson et al., 2021; USFWS, 2023, 2024).

## FINANCIAL FEASIBILITY AND CHANGE IN ABSOLUTE AND RELATIVE COSTS WITH ECOLOGICAL CHANGE

Every natural resources manager works under budgetary constraints, which inevitably influence management choices and often require making difficult trade‐offs in the face of multiple or even conflicting priorities (Ellenswood et al., 2012). Appropriated budgets tend to remain static and may be degraded by inflation or, occasionally, increased in response to changing policy priorities. Other revenue (e.g., grazing fees, entrance fees) is based on formulas that are slow to change in response to economic and environmental conditions and are more or less fungible, depending on how they are directed (e.g., to maintenance, specific program areas, or general funds) (Loomis, 1993). Most natural resource managers do not operate in a classical or even regulated market but in something closer to pure public finance and public goods (Hackett, 1998). Here, the mandate may be to provide public access and services (setting fees for some services with few market signals as guides), attend to distributional and equity concerns, and meet the expectations of the public, such as particular types of recreational experiences. Nevertheless, some microeconomic principles come to bear on management choices, such as the role of costs in driving innovation or ways to achieve economies of scale as ecological transformations proceed.

The relative financial feasibility of alternative management approaches changes over time, and managers in a given unit generally have little influence on the larger economic and policy forces that drive those changes. Some costs, such as the aggregate costs of wildfire suppression, tend to increase over time (Gebert et al., 2007), driven by factors like increasing labor and equipment costs (Canton‐Thompson et al., 2006). Technological inputs could play an increasing role in the future and may offset certain costs (Canton‐Thompson et al., 2006). For instance, drones may increase use of aerial resources with less equipment or labor costs, and earth science data streams may reduce the costs of in situ monitoring.

Nevertheless, natural resource managers have faced tight budgets for a long time in the United States (Leshy, 2021) and likely will continue to do so into the future, which means most units cannot afford new expensive interventions and lean toward cost‐effective strategies (i.e., getting the best outcome given a fixed budget). A possible exception to this is that in public finance settings, funds may flow to dire needs. For instance, the rising costs of suppressing increasingly frequent and severe wildfires, especially as the wildland–urban interface expands (Burke et al., 2021; Radeloff et al., 2023), have led to recent attempts to address past budget mismatches between needs and available funds (e.g., Steffens, 2023; U.S. Senate, 2023; USFS, 2022).

Ecological transformation will alter the absolute and relative costs of RAD strategies. Investment in resist strategies increases with environmental change as external forces push systems further from desired current or historic condition. The opportunity costs (foregoing the potential benefit of the next best alternative) of resist strategies may also increase over time. In other words, as resisting becomes more costly, the trade‐off between spending money to resist (e.g., expanded firefighting), perhaps with less certain chances of success, and spending on allied objectives (e.g., community fire risk mitigation) grows (Schoennagel et al., 2017). Some resist strategies will remain or become financially prohibitive, for instance, propagating coral reefs (Madin et al., 2019) or protecting melting glaciers from increasing ablation (Senese et al., 2020; Huss et al., 2021). Accept strategies may be considered to require fewer financial inputs because they involve less intervention, although they are likely to yield reduced resource output for most significant ecological transformations. We believe the greatest uncertainty lies with costs to direct ecological transitions. Within the broad swath of uncertain feasibility, the most cost‐efficient and financially feasible direct strategies would work with current or emerging social–ecological trajectories to steer and nudge systems into resilient states that can largely function without regular management intervention while still providing desirable ecological services.

**FIGURE 3:**
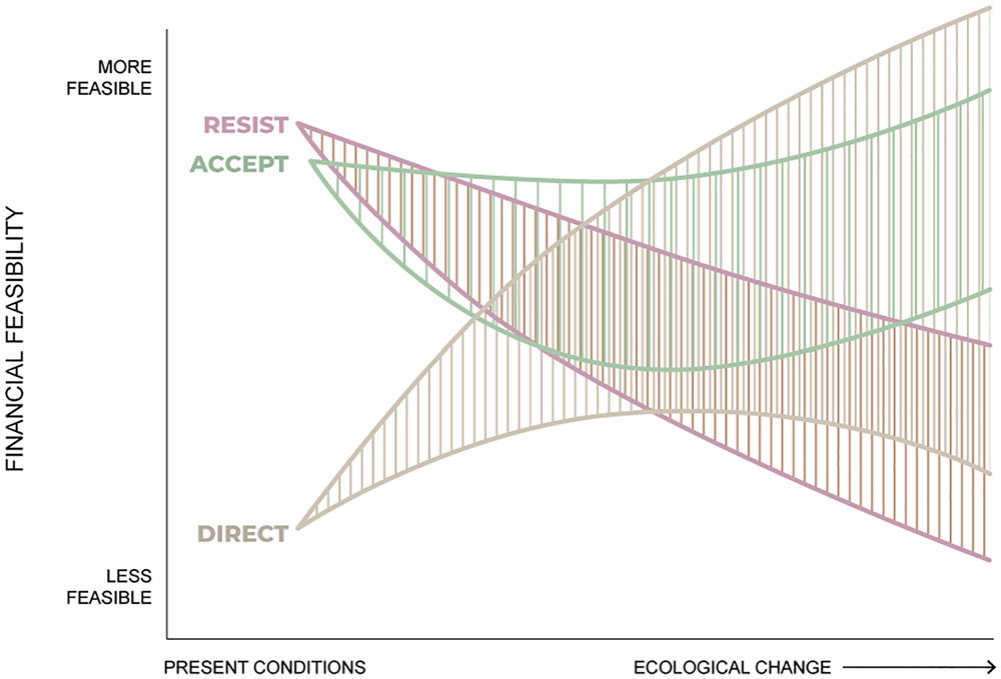
How financial feasibility of resist, accept, and direct strategies might evolve over time.

In Figure 3, our financial feasibility storylines start with resist and accept strategies on a roughly equal footing, both declining with continuing ecological transformation, but accept strategies recover feasibility as managers and stakeholders tolerate altered ecological services (Smith et al., 2022) and relax demands for costly resistance. Resist feasibility continues to decline throughout our hypothetical planning frame. We posit that costs to direct change may decrease over time due to technological innovation and learning, the latter especially focused on applying new insights and monitoring to nudge systems toward satisfactory desired states. We retained a wide feasibility space for direct strategies to represent the cost of ongoing intervention that may keep some options from implementation. In Figure 3, curves represent one set of possible future trajectories and account for uncertainty about more distant futures.

## PUBLIC FEASIBILITY, CHANGES IN STAKEHOLDER ATTITUDES, AND SHIFTING DECISION‐MAKING CONSTRAINTS

Natural resource laws require managers to be responsive to the preferences and expectations of a range of stakeholders when making decisions, including resource users, surrounding communities, businesses, local and state government, and citizens across the nation (Biggs et al., 2011; Vogler et al., 2017). Many factors shape preferences, including worldviews, social norms, political economic forces, personal experience, and identity (Bengston, 1994; Bennett et al., 2017; Clifford et al., 2022). The public influences decisions both through formal (e.g., public comments) and informal avenues (e.g., political pressure, behavioral patterns when recreating). Legal action against agencies creates an additional impetus to consider public attitudes, at least in the United States (Martin, 2021; Sayre, 2006). Beyond formal mechanisms, stakeholder and public pressure can shape managers’ decisions because they perceive and have a responsibility to serve the public, live as neighbors in communities surrounded by public lands, and are susceptible to political pressure. Such influences can constrain which management strategies are politically possible, referring to the recognition that “compromise, stakeholder engagement, institutional realities, and an awareness of costs and benefits combine to structure” feasibility (Wilson et al., 2018, p. 1). Public feasibility is the way that managers understand stakeholder and public attitudes as a (perceived or actual) constraint on decision‐making. Public attitudes and expectations about landscapes, their management, and the appropriate way to respond to environmental change evolve over time and are shaped by interactions and feedbacks with other types of feasibility. The way these attitudes shape and constrain manager decision‐making will similarly evolve over time in conjunction with other types of feasibility. As a result, public views will be an important factor across strategies, and managers will need to engage with them in meaningful ways to avoid conflict and friction (Magness et al., 2022).

Just as the factors shaping the public and stakeholders’ preferences for natural resource management are multiple and complex, so too are the factors that lead people to change their attitudes over time. Public views will vary considerably based on a number of factors unique to individuals and stakeholder groups and will never be fully in agreement, but one can expect some broad trends in opinion, alongside a continued diversity of viewpoints. Learning (Heikkila & Gerlak, 2013; Gerlak et al., 2018), social movements (Earl, 2004; Wilson, 1997), political affiliations and polarization (Leiserowitz et al., 2019), legacies of conflicts (Colven et al., 2015), shifts in livelihoods (Persha et al., 2011, 2010), developing or affirming new aspects of one's identity (Kosek, 2004; Barnett et al., 2021), and changing social norms (Minato et al., 2010; Nyborg et al., 2016) can all lead people to shift their opinions. No one theory can broadly explain shifts in attitudes, and social scientists disagree about which factors have greater explanatory power (Heberlein, 2012). This is because changes in beliefs, attitudes, and preferences over time occur at individual and collective scales and at slow and rapid paces. For example, social movements—often led by charismatic individuals—have substantially shifted people's attitudes about natural resources. In the 1960s, Rachel Carson and the environmental movement led to significant shifts in American's attitudes about nature, and that public support led to a flush of landmark legislation that sought to protect the environment (Kline, 2022). Twenty years later, the Wise Use Movement galvanized rural communities across the American West and led to widespread shifts in attitudes that emphasized private property and nature as resource (Echeverria & Eby, 1995).

Despite the presence of the factors that can drive change, in many cases public attitudes and opinions persist. For instance, new information and even personal experiences with climate impacts do not necessarily lead to learning or changed beliefs about climate change. Political affiliation affects how people think about climate change (Leiserowitz et al., 2019), and this might either shift beliefs or solidify them. Despite this complexity, there can be clues about how opinions are shifting over time through public opinion polls (e.g., Leiserowitz et al., 2019). It can be expected that preferences and attitudes that influence natural resource management will shift over time, but how, when, and why cannot be fully anticipated.

As public views evolve, the public feasibility of different types of RAD strategies will also change. In the near term, resist strategies are comparatively likely to face few objections because they are familiar and fit within traditional natural resource paradigms (i.e., maintaining baseline conditions). In the longer term, however, resist strategies may be increasingly controversial due to rising costs, unintended consequences, or perceptions of being ecologically or technically impossible (e.g., preventing glaciers melt). Accept strategies will likely be less favorable to the public in the near term because they could be viewed as giving up (e.g., accepting invasive species). An exception to this might be in wilderness, where accept may be seen as consistent with the mandate to leave land “untrammeled” (Public Law 88‐577). Finally, direct strategies are likely to be the least publicly feasible in the short term because they are likely to be perceived as unprecedented or high risk (Sjoberg, 2000), carrying the potential for many unintended consequences (e.g., introducing new species) (Hagerman & Satterfield, 2014; Scott, 2023). Stakeholders and the public are likely to perceive such heavy‐handed or untested approaches as unwarranted or high risk until ecological trajectories proceed to a point of significant, visible, and perhaps irreversible change (St‐Laurent et al., 2018). However, direct strategies will likely become more publicly feasible if stakeholders and the public increasingly come to view ecological transformation as a serious threat or see examples of successful direct experiments (e.g., Nadeau et al., 2024; Crausbay et al., 2022; Miller‐Rushing & Henkel, 2022). Of course, determining when transformation is happening or has occurred is a difficult task due to shifting baselines and because different individuals or communities may have different internal thresholds and lenses through which they understand change (Datta et al., 2024).

**FIGURE 4:**
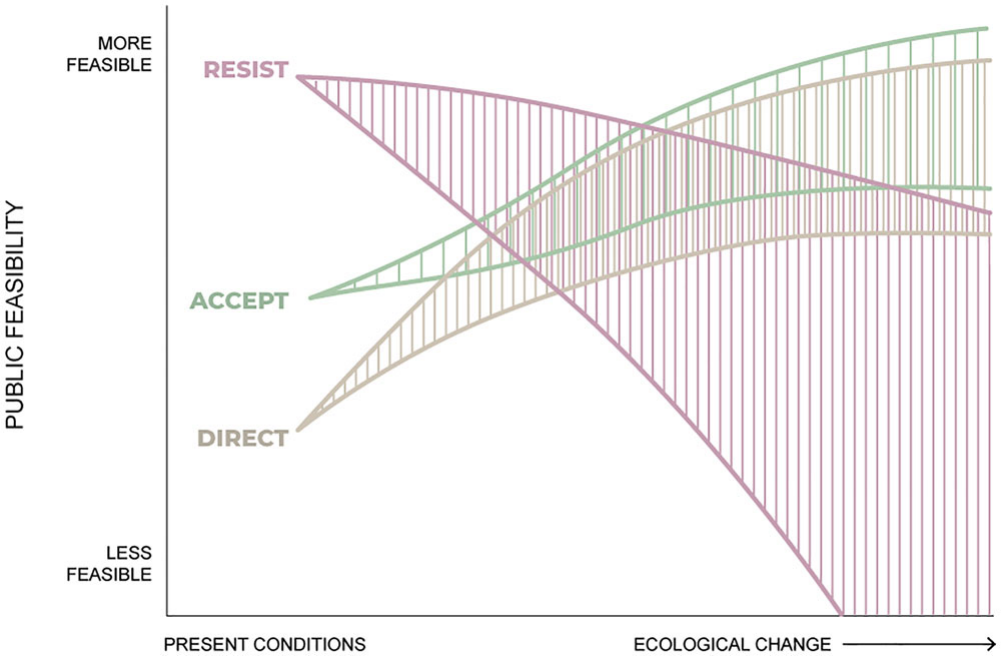
How public feasibility of resist, accept, and direct strategies might evolve over time.

In Figure 4, as citizen and stakeholder attitudes evolve in response to changing ecological conditions, we expect that accept and direct strategies will become more viable, with direct strategies being at an early disadvantage given the widespread distrust at present of meddling with nature (Hagerman & Satterfield, 2014; Sjoberg, 2000). Conversely, resist strategies may become a less preferred option (and thus less publicly feasible) as resist strategies become more ecologically challenging or expensive (Millar et al., 2007; St‐Laurent et al., 2018). We greatly expanded the range of uncertainty of resist strategies to encompass the possibility of emerging public support for direct strategies that involve higher levels of intervention in ecosystem process and function. In Figure 4, curves represent one set of possible future trajectories and account for uncertainty about more distant futures.

## ORGANIZATIONAL FEASIBILITY AND THE EVOLUTION OF MANAGERS’ INDIVIDUAL AND UNIT‐LEVEL PERSPECTIVES

Natural resource managers hold a significant amount of discretion in most decision‐making situations, subject to the constraints created by law and policy (i.e., regulatory feasibility), best available scientific information (e.g., Francis et al., 2005), available funding (i.e., financial feasibility), and stakeholder and public expectations (i.e., public feasibility) (Clifford et al., 2022; Ellenwood et al., 2012). As one study of attitudes toward forest management for wildfire prevention concluded, “‘within‐the‐ranks’ professional resistance [may] be more of a factor affecting the implementation of fire management policies than public resistance… Policies that cause organizations to rethink their basic mission and initiate new ways of implementing programs are often resisted by those responsible for implementation” (Gardner et al., 1985, p. 309). A variety of factors, including increased recognition of diverse ways of valuing nature (Pascual et al., 2023), are forcing managers to shift the way they think about management. Managers’ (individual or collective) understanding of a strategy as possible or desirable is termed organizational feasibility.

Natural resource managers’ perspectives are influenced by their mental models, which are conceptual representations of the world that people use to solve problems they encounter (Denzau & North, 1994; Jones et al., 2011). Individuals’ mental models are shaped by their worldviews (including identity, sense of place, values, and attitudes), the cultures they belong to, and their understanding of social and environmental systems (Clifford et al., 2022). Each of these elements can change over time, leading to evolution of that model. First, aspects of worldviews motivate how people act in the world but can also transform as people encounter new situations or make sense of new experiences. For instance, one's sense of place can contribute to stewardship actions (Chapin & Knapp, 2015), but rapid changes in an environmental system can also shift how one perceives and responds to place (Oakes et al., 2016).

Second, the culture of a manager's unit, agency, community, and society at large influences their mental model and ultimately their perspective on which strategies seem possible or desirable (Page & Dilling 2020). Individual perspectives interact and aggregate to influence how organizations understand and evaluate the feasibility of choices (Maitlis & Christianson, 2014; Weick, 1995). For example, individual risk perception may interact with organizational risk perception to influence which options managers see as too risky (Cole et al., 2022). Accepting the potential for wildfire may seem less risky if there is organizational support and encouragement for allowing natural fire regimes. If organizational risk perception increases due to increased wildland–urban interface development, managers may see this option as less feasible.

Finally, managers’ understanding of how institutional and ecological systems function shapes which actions they see as possible or preferable. This understanding may evolve as new information becomes available (Crausbay et al., 2022), as they develop new explanations for cause‐and‐effect, or as they encounter unprecedented situations, such as an extreme drought or 1‐in‐500‐year flood event, that challenge their mental model (e.g., Carlton et al., 2016; Koontz & Miller, 2022; Poland et al., 2022). Attitudes and beliefs about natural systems can similarly shift gradually based on long‐term, intimate observation and interaction in particular locations (e.g., Knapp & Fernandez‐Gimenez, 2009; Knapp et al., 2014), although such shifts appear to be mediated by knowledge and perception of underlying causes (Oakes et al., 2016). Perceptions of organizational systems (e.g., decision‐making processes or valued workforce skills and aptitudes) can also influence what seems feasible. For example, lack of workforce skills in newer remote‐sensing monitoring practices may make the use of a direct straegy seem more risky if there is not adequate monitoring to assess whether actions are effective. In addition, formal learning experiences, such as recent scenario planning workshops for the National Park Service (Miller et al., 2022), may change how managers perceive systems. Learning theory and the learning loop framework (Argyris & Schön, 1974, 1996) suggests that the process of making RAD decisions will itself result in evolutions in mental models over time as managers learn by doing (Fabricius & Cundill, 2014). In the case of RAD decision‐making, loop learning is propelled by a mismatch between the ecological conditions agencies or managers observe and the management tools and paradigms they employ or constraints they face (Lynch et al., 2022).

**FIGURE 5:**
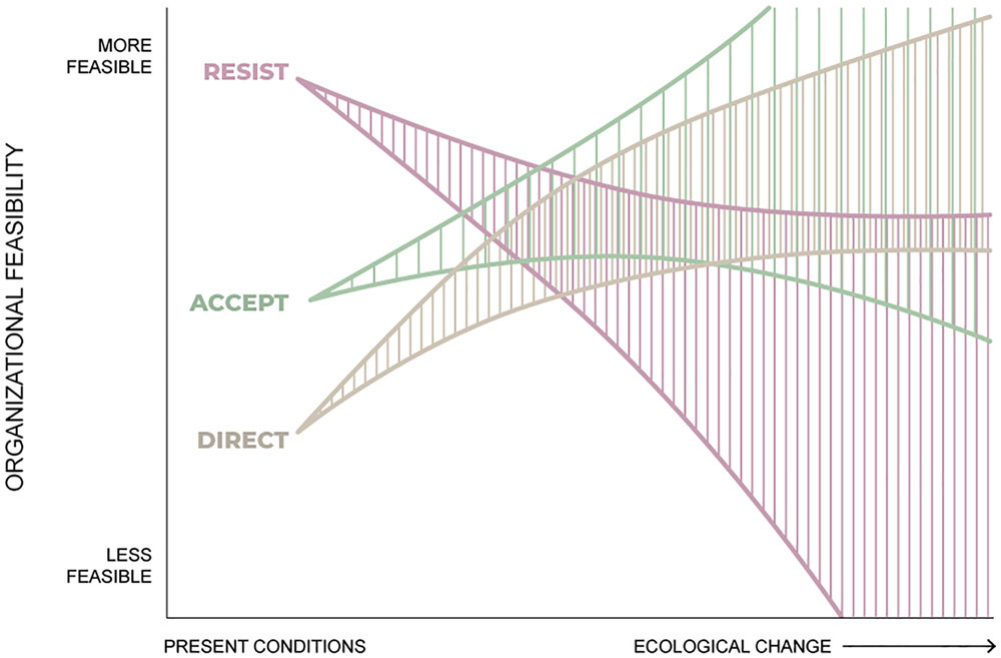
How organizational feasibility of resist, accept, and direct strategies might evolve over time.

The RAD framework represents an ongoing paradigm shift for many natural resource management agencies (Schuurman et al., 2022), leading to shifts in the organizational feasibility of various options (Figure 5). Historically, resist strategies were familiar and perceived as more feasible because they were consistent with managers’ mental models. As individuals and agencies encounter experiences where resisting failed (as happened in the case of the endangered Karner blue butterfly [*Plebejus melissa samuelis*] in Indiana Dunes National Park [Schuurman et al., 2023]) and the stakes of what is threatened increase, resist strategies may come to seem increasingly impractical, prompting learning within teams or agencies that results in decreased organizational feasibility for resist strategies. For instance, invasive species may cross a threshold of landscape cover where attempts to control spread are no longer viewed as feasible, and managing with the invasive becomes normalized. Similarly, direct strategies, such as conservation translocation, have to date been uncommon and seen as options of last resort (Cole et al., 2022), such as is happening with native bird management in the Pacific islands (Paxton et al., 2022). Organizational feasibility of any given future effort to direct change will be determined by manager perceptions and learning from prior direct actions. Some managers see accepting strategies as a natural evolution of ecological change (Cole & Yung, 2010) and may continue to do so over time. For instance, managers may allow natural disturbance cycles, such as fire, to continue despite the fact that climate change has shifted the intensity and extent of historical fire behavior. Others may change their opinions once ecological conditions change so much that a place no longer has the same sense of place or supports the same activities. The result would be decreased organizational feasibility for accept. Additionally, accepting strategies may be seen as a safe choice when uncertainty is high, but as examples of crossing ecological thresholds increase and become well known, managers may come to view accepting strategies as more risky and thus less organizationally feasible than they appear at present.

In Figure 5, as managers gain experience and learn, their individual and collective perspectives on managing ecological transformation will evolve. We expect that resist strategies will become less feasible as managers encounter situations where resisting is ecologically, by regulation, financially, or publicly not feasible (e.g., Schuurman et al., 2023). Similarly, we expect that as regulatory frameworks increasingly permit them, if costs decrease, and if the public accepts them, managers will increasingly view accept and direct strategies as viable options. In Figure 5, we greatly expanded the range of uncertainty for resist strategies to encompass the possibility that, although it declines, attitude changes toward aggressive forms of resist‐oriented interventions might give managers wider remit to experiment with bending transformation back toward familiar territory, an option less imaginable for accept and direct. In Figure 5, curves represent one set of possible future trajectories and account for uncertainty about more distant futures.

## CONCLUSION

The decisions that managers face in response to ecological transformations are dynamic and complex. Exploring how elements of feasibility shift over time enables better understanding of potential pathways that might emerge from different sets of choices and how managers might commit themselves to different trajectories. Exploring these potential trajectories requires adoption and application of a linked socioecological framework and a transdisciplinary approach in partnership with managers to understand, manage, and learn from change. The reality of ecological transformation necessitates a more integrative approach with social science in order to better understand how social responses influence ecological outcomes and vice versa. We argued that the social feasibility of RAD decisions has multiple elements that vary in response to distinct social causes and drivers (Figure 1c), thereby moving beyond previous conceptions that lumped these elements under the umbrella of “social feasibility” (Lynch et al., 2021) (Figure 1b) as well as expanding on a conceptual framework of managers’ decision spaces (Clifford et al., 2022) (Figure 1a), which only considered a single point in time. Social response to ecological transformation is complex and will influence pathways of ecological transformation through socioecological feedbacks (Blythe et al., 2017) and response pathways (Thaler et al., 2023). In addition, responding effectively to ecological transformation will require continuous learning and adapting, suggesting the importance of small‐scale experiments to empirically test, adapt, and scale up strategies in real‐world contexts, as is happening in Acadia National Park (Nadeau et al., 2024).

Because each decision that is made over time affects future social and ecological trajectories, shapes future decisions, and potentially sets decision pathways (Colloff et al., 2017), it is important to think about what managers and society will choose and when they will choose it. Although some decisions will be gradual, others might arise in distinct windows of opportunity. Concerns of ecological catastrophe may lead to short‐lived allocations of funding, and RAD planning in these contexts might effectively and efficiently utilize these extra funds to transition from resist to accept or direct strategies. For example, increased wildfire activity and increasingly severe fires led in recent years to reassessment of funding for US wildfire agencies (e.g. Crowley et al., 2023). Tools, such as scenario planning and other approaches to futures thinking, may become increasingly important as time horizons for decision‐making expand (e.g., Coulter et al., 2019).

We focused on how incremental ecological transformation might influence changes in the feasibility of different RAD strategies over time. We assumed the degree of ecological transformation and time are roughly correlated (Figures 2, 3, 4, 5), but ecological transformation can be incremental or abrupt (Williams et al., 2021). Abrupt transformation will be driven by threshold changes in ecological conditions that would lead to sudden shifts, with potentially drastic consequences for social systems (e.g., Willcock et al., 2023) and perceived feasibility of options. Future research could examine the shifting feasibility of RAD decision‐making in contexts of abrupt transformation. We only hinted at abrupt social changes that could influence feasibility of options, but we recognize that technological breakthroughs, pandemic‐scale health challenges, radical political shifts, or violent social conflict could similarly have a transformative effect on the factors and feasibility of the strategies we considered (e.g., Smith et al., 2022). It is important to stay aware of nonlinear feedbacks and events in social and ecological systems that may influence feasibility of RAD decisions.

Society faces the monumental task of anticipating, preparing for, and responding to novel conditions that are transforming ecological systems. Uncertainty about future trajectories and the outcomes of manager responses will be intimately connected to social, political, economic, and cultural systems. Recognizing the dynamic nature of social trajectories and considering feedbacks between social and ecological changes is an important step to making natural resource decisions in a dynamic, uncertain world.

## AUTHOR CONTRIBUTIONS

Amanda E. Cravens, Katherine R. Clifford, and Corrine Knapp conceptualized the project, and all authors wrote the original draft, revised and edited the manuscript, and approved the final version for publication.

## References

[cobi14331-bib-0001] Argyris, C. , & Schön, D. (1974). Theory in practice. Jossey‐Bass.

[cobi14331-bib-0002] Argyris, C. , & Schön, D. (1996). Organizational learning II. Addison‐Wesley.

[cobi14331-bib-0003] Barnett, J. , Graham, S. , Quinn, T. , Adger, W. N. , & Butler, C. (2021). Three ways social identity shapes climate change adaptation. Environmental Research Letters, 16(12), Article 124029. 34840601 10.1088/1748-9326/ac36f7 PMC8611257

[cobi14331-bib-0004] Bates, A. E. , Stuart‐Smith, R. D. , Barrett, N. S. , & Edgar, G. J. (2017). Biological interactions both facilitate and resist climate‐related functional change in temperate reef communities. Proceedings of the Royal Society B: Biological Sciences, 284(1856), Article 20170484. 10.1098/rspb.2017.0484 PMC5474073 28592671

[cobi14331-bib-0005] Bengston, D. N. (1994). Changing forest values and ecosystem management. Society & Natural Resources, 7(6), 515–533.

[cobi14331-bib-0006] Bennett, N. J. , Roth, R. , Klain, S. C. , Chan, K. , Christie, P. , Clark, D. A. , Cullman, G. , Curran, D. , Durbin, T. J. , Epstein, G. , & Wyborn, C. (2017). Conservation social science: Understanding and integrating human dimensions to improve conservation. Biological Conservation, 205, 93–108.

[cobi14331-bib-0007] Biggs, D. , Abel, N. , Knight, A. T. , Leitch, A. , Langston, A. , & Ban, N. C. (2011). The implementation crisis in conservation planning: Could “mental models” help? Conservation Letters, 4(3), 169–183.

[cobi14331-bib-0008] Biggs, R. , Peterson, G. D. , & Rocha, J. C. (2018). The Regime Shifts Database: A framework for analyzing regime shifts in social‐ecological systems. Ecology and Society, 23(3), Article 9.

[cobi14331-bib-0009] Blythe, J. , Nash, K. , Yates, J. , & Cumming, G. (2017). Feedbacks as a bridging concept for advancing transdisciplinary sustainability research. Current Opinion in Environmental Sustainability, 26, 114–119.

[cobi14331-bib-0010] Burke, M. , Driscoll, A. , Heft‐Neal, S. , Xue, J. , Burney, J. , & Wara, M. (2021). The changing risk and burden of wildfire in the United States. Proceedings of the National Academy of Sciences of the United States of America, 118(2), Article e2011048118. 33431571 10.1073/pnas.2011048118 PMC7812759

[cobi14331-bib-0011] California Ocean Protection Council . (2018). State of California Sea‐Level Rise Guidance, 2018 Update . https://www.opc.ca.gov/updating‐californias‐sea‐level‐rise‐guidance/

[cobi14331-bib-0012] Canton‐Thompson, J. , Thompson, B. , Gebert, K. M. , Calkin, D. E. , Donovan, G. H. , & Jones, G. (2006). Factors affecting fire suppression costs as identified by incident management teams (Research Note RMRS‐RN‐30). USDA Forest Service, Rocky Mountain Research Station.

[cobi14331-bib-0013] Carlton, J. S. , Mase, A. S. , Knutson, C. L. , Lemos, M. C. , Haigh, T. , Todey, D. P. , & Prokopy, L. S. (2016). The effects of extreme drought on climate change beliefs, risk perceptions, and adaptation attitudes. Climatic Change, 135, 211–226.

[cobi14331-bib-0014] Chapin, F. S. , & Knapp, C. N. (2015). Sense of place: A process for identifying and negotiating potentially contested visions of sustainability. Environmental Science & Policy, 53, 38–46.

[cobi14331-bib-0015] Christensen, M. R. , Graham, M. D. , Vinebooke, R. D. , Findlay, D. L. , Paterson, M. J. , & Turner, M. A. (2006). Multiple anthropogenic stressors cause ecological surprises in boreal lakes. Global Change Biology, 12(12), 2316–2322.

[cobi14331-bib-0016] Christensen, N. L. , Bartuska, A. M. , Brown, J. H. , Carpenter, S. , d'Antonio, C. , Francis, R. , Franklin, J. F. , MacMahon, J. A. , Noss, R. F. , Parsons, D. J. , & Peterson, C. H. (1996). The report of the Ecological Society of America committee on the scientific basis for ecosystem management. Ecological Applications, 6(3), 665–691.

[cobi14331-bib-0017] Clifford, K. R. , Cravens, A. E. , & Knapp, C. N. (2022). Responding to ecological transformation: Mental models, external constraints, and manager decision‐making. Bioscience, 72(1), 57–70.

[cobi14331-bib-0018] Clifford, K. R. , Yung, L. , Travis, W. R. , Rondeau, R. , Neely, B. , Rangwala, I. , Burkardt, N. , & Wyborn, C. (2020). Navigating climate adaptation on public lands: How views on ecosystem change and scale interact with management approaches. Environmental Management, 66, 614–628. 32728791 10.1007/s00267-020-01336-y PMC7522104

[cobi14331-bib-0019] Cole, D. N. , & Yung, L. (2010). Beyond naturalness: Rethinking park and wilderness stewardship in an era of rapid change. Island Press.

[cobi14331-bib-0020] Cole, N. , Goolsby, J. B. , & Cravens, A. E. (2022). Perceptions of conservation introduction to inform decision support among U.S. Fish and Wildlife Service employees: U.S. Geological Survey Scientific Investigations Report 2022–5092 . U.S. Geological Survey. 10.3133/sir20225092

[cobi14331-bib-0021] Colloff, M. J. , Lavorel, S. , van Kerkhoff, L. E. , Wyborn, C. A. , Fazey, I. , Gorddard, R. , Mace, G. M. , Foden, W. B. , Dunlop, M. , Prentice, I. C. , & Crowley, J. (2017). Transforming conservation science and practice for a postnormal world. Conservation Biology, 31(5), 1008–1017. 28225163 10.1111/cobi.12912

[cobi14331-bib-0022] Colvin, R. M. , Witt, G. B. , & Lacey, J. (2015). The social identity approach to understanding socio‐political conflict in environmental and natural resources management. Global Environmental Change, 34, 237–246.

[cobi14331-bib-0023] Coulter, L. , Serrao‐Neumann, S. , & Coiacetto, E. (2019). Climate change adaptation narratives: Linking climate knowledge and future thinking. Futures, 111, 57–70.

[cobi14331-bib-0024] Council on Environmental Quality (CEQ) . (2023). Biden‐⁠Harris administration releases new guidance to disclose climate impacts in environmental reviews. The White House. https://www.whitehouse.gov/ceq/news‐updates/2023/01/06/biden‐harris‐administration‐releases‐new‐guidance‐to‐disclose‐climate‐impacts‐in‐environmental‐reviews/

[cobi14331-bib-0025] Crausbay, S. D. , Sofaer, H. R. , Cravens, A. E. , Chaffin, B. C. , Clifford, K. R. , Gross, J. E. , Knapp, C. N. , Lawrence, D. J. , Magness, D. R. , Miller‐Rushing, A. J. , Schuurman, G. W. , & Stevens‐Rumann, C. S. (2022). A science agenda to inform natural resource management decisions in an era of ecological transformation. Bioscience, 72(1), 71–90.

[cobi14331-bib-0026] Crowley, C. , Miller, A. , Richardson, R. , & Malcom, J. (2023). Increasing damages from wildfires warrant investment in wildland fire management (Report R‐2023‐01). Office of Policy Analysis, U.S. Department of the Interior.

[cobi14331-bib-0027] Datta, A. W. , Wyborn, C. , Chaffin, B. C. , & Barnes, M. L. (2024). Imagining reef futures after mass coral bleaching events. Environmental Science & Policy, 151, Article 103625.

[cobi14331-bib-0028] DeCaro, D. A. , Chaffin, B. C. , Schlager, E. , Garmestani, A. S. , & Ruhl, J. B. (2017). Legal and institutional foundations of adaptive environmental governance. Ecology and Society: A Journal of Integrative Science for Resilience and Sustainability, 22(1), 1–32. 10.5751/ES-09036-220132 PMC5954432 29780428

[cobi14331-bib-0029] Denzau, A. T. , & North, D. C. (1994). Shared mental models: Ideologies and institutions. Kyklos, 47(1), 3–31.

[cobi14331-bib-0030] Earl, J. (2004). The cultural consequences of social movements. In D. A. Snow , S. A. Soule , & K. Hanspeter (Eds.), The Blackwell companion to social movements (pp. 508–530). Wiley.

[cobi14331-bib-0031] Echeverria, J. D. , & Eby, R. B. (Eds.). (1995). Let the people judge: Wise use and the private property rights movement. Island Press.

[cobi14331-bib-0032] Ellenwood, M. S. , Dilling, L. , & Milford, J. B. (2012). Managing United States public lands in response to climate change: A view from the ground up. Environmental Management, 49, 954–967. 22437431 10.1007/s00267-012-9829-2

[cobi14331-bib-0033] Endangered Species Act, Public Law 93–205, 87 Stat. 884, 16 U.S.C. §§1531‐1544 (1973).

[cobi14331-bib-0034] Fabricius, C. , & Cundill, G. (2014). Learning in adaptive management: Insights from published practice. Ecology and Society, 19(1), Article 29.

[cobi14331-bib-0035] Fidelman, P. , McGrath, C. , Newlands, M. , Dobbs, K. , Jago, B. , & Hussey, K. (2019). Regulatory implications of coral reef restoration and adaptation under a changing climate. Environmental Science & Policy, 100, 221–229.

[cobi14331-bib-0036] Fortini, L. B. , Kaiser, L. R. , & LaPointe, D. A. (2020). Fostering real‐time climate adaptation: Analyzing past, current, and forecast temperature to understand the dynamic risk to Hawaiian honeycreepers from avian malaria. Global Ecology and Conservation, 23, Article e01069.

[cobi14331-bib-0037] Francis, T. B. , Whittaker, K. A. , Shandas, V. , Mills, A. V. , & Graybill, J. K. (2005). Incorporating science into the environmental policy process: A case study from Washington State. Ecology and Society, 10(1), Article 35. http://www.ecologyandsociety.org/vol10/iss1/art35/

[cobi14331-bib-0038] Gardner, P. D. , Cortner, H. J. , Widaman, K. F. , & Stenberg, K. J. (1985). Forest‐user attitudes toward alternative fire management policies. Environmental Management, 9, 303–311.

[cobi14331-bib-0039] Gebert, K. M. , Calkin, D. E. , & Yoder, J. (2007). Estimating suppression expenditures for individual large wildland fires. Western Journal of Applied Forestry, 22(3), 188–196.

[cobi14331-bib-0040] Gerlak, A. K. , Heikkila, T. , Smolinski, S. L. , Huitema, D. , & Armitage, D. (2018). Learning our way out of environmental policy problems: A review of the scholarship. Policy Sciences, 51, 335–371.

[cobi14331-bib-0041] Gupta, J. , Termeer, C. , Klostermann, J. , Meijerink, S. , Van den Brink, M. , Jong, P. , Nooteboom, S. , & Bergsma, E. (2010). The adaptive capacity wheel: A method to assess the inherent characteristics of institutions to enable the adaptive capacity of society. Environmental Science & Policy, 13(6), 459–471.

[cobi14331-bib-0042] Hackett, S. C. (1998). Environmental and natural resources economics: Theory, policy, and the sustainable society. M.E. Sharpe.

[cobi14331-bib-0043] Hagerman, S. M. , & Satterfield, T. (2014). Agreed but not preferred: Expert views on taboo options for biodiversity conservation, given climate change. Ecological Applications, 24(3), 548–559. 24834740 10.1890/13-0400.1

[cobi14331-bib-0044] Hamlin, A. (2017). Feasibility four ways. Social Philosophy and Policy, 34(1), 209–231.

[cobi14331-bib-0045] Heberlein, T. A. (2012). Navigating environmental attitudes. Oxford University Press. 10.1111/j.1523-1739.2012.01892.x 22809349

[cobi14331-bib-0046] Heikkila, T. , & Gerlak, A. K. (2013). Building a conceptual approach to collective learning: Lessons for public policy scholars. Policy Studies Journal, 41(3), 484–512.

[cobi14331-bib-0130] Huss, M. , Schwyn, U. , Bauder, A. , & Farinotti, D. (2021). Quantifying the overall effect of artificial glacier melt reduction in Switzerland, 2005–2019. Cold Regions Science and Technology, 184, 103237.

[cobi14331-bib-0048] Jenny, J. P. , Anneville, O. , Arnaud, F. , Baulaz, Y. , Bouffard, D. , Domaizon, I. , Bocaniov, S. A. , Chèvre, N. , Dittrich, M. , Dorioz, J.‐M. , Dunlop, E. S. , Dur, G. , Guillard, J. , Guinaldo, T. , Jacquet, S. , Jamoneau, A. , Jawed, Z. , Jeppesen, E. , Krantzberg, G. , & Weyhenmeyer, G. A. (2020). Scientists’ warning to humanity: Rapid degradation of the world's large lakes. Journal of Great Lakes Research, 46(4), 686–702.

[cobi14331-bib-0050] Jones, N. A. , Ross, H. , Lynam, T. , Perez, P. , & Leitch, A. (2011). Mental models: An interdisciplinary synthesis of theory and methods. Ecology and Society, 16(1), Article 46. 10.5751/ES-03802-160146

[cobi14331-bib-0051] Judge, S. W. , Warren, C. C. , Camp, R. J. , Berthold, L. K. , Mounce, H. L. , Hart, P. J. , & Monello, R. J. (2021). Population estimates and trends of three Maui Island‐endemic Hawaiian honeycreepers. Journal of Field Ornithology, 92(2), 115–126.

[cobi14331-bib-0052] Karasov‐Olson, A. , Schwartz, M. W. , Olden, J. D. , Skikne, S. , Hellmann, J. J. , Allen, S. , Brigham, C. , Buttke, D. , Lawrence, D. J. , Miller‐Rushing, A. J. , & Morisette, J. T. , & Hawkins‐Hoffman, C. (2021). Ecological risk assessment of managed relocation as a climate change adaptation strategy (Natural Resource Report 2021/2241). National Park Service. 10.36967/nrr-2284919

[cobi14331-bib-0053] Kline, B. (2022). First along the river: A brief history of the US environmental movement. Rowman & Littlefield.

[cobi14331-bib-0054] Knapp, C. N. , Chapin III, F. S. , Kofinas, G. P. , Fresco, N. , Carothers, C. , & Craver, A. (2014). Parks, people, and change: The importance of multistakeholder engagement in adaptation planning for conserved areas. Ecology and Society, 19(4), Article 16. 10.5751/ES-06906-190416

[cobi14331-bib-0055] Knapp, C. N. , & Fernandez‐Gimenez, M. (2009). Knowledge in practice: Exploring rancher's ecological knowledge in NW Colorado. Rangeland Ecology and Management, 62(6), 500–509.

[cobi14331-bib-0056] Koontz, H. K. , & Miller, C. E. (2022). USGS Media Alert: USGS crews continue to measure and assess Yellowstone River flood conditions and probabilities. U.S. Geological Survey. https://www.usgs.gov/news/state‐news‐release/usgs‐media‐alert‐usgs‐crews‐continue‐measure‐and‐assess‐yellowstone‐river

[cobi14331-bib-0057] Kosek, J. (2004). Deep roots and long shadows: The cultural politics of memory and longing in northern New Mexico. Environment and Planning D: Society and Space, 22(3), 329–354.

[cobi14331-bib-0058] Larrosa, C. , Carrasco, L. R. , & Milner‐Gulland, E. J. (2016). Unintended feedbacks: Challenges and opportunities for improving conservation effectiveness. Conservation Letters, 9(5), 316–326.

[cobi14331-bib-0059] Leiserowitz, A. , Maibach, E. , Rosenthal, S. , Kotcher, J. , Bergquist, P. , Ballew, M. , Goldberg, M. , & Gustafson, A. (2019). Climate change in the American mind: November 2019. Yale University and George Mason University, Yale Program on Climate Change Communication.

[cobi14331-bib-0060] Leshy, J. D. (2021). Our common ground: A history of America's public lands. Yale University Press.

[cobi14331-bib-0061] Lieberman, L. , Hahn, B. , & Landres, P. (2018). Manipulating the wild: A survey of restoration and management interventions in US wilderness. Restoration Ecology, 26(5), 900–908.

[cobi14331-bib-0062] Lieberman, L. A. (2017). The balancing act: Ecological interventions and decision tradeoffs to preserve wilderness character [Unpublished master's thesis]. University of Montana.

[cobi14331-bib-0063] Loomis, J. B. (1993). Integrated public lands management: Principles and applications to national forests, parks, wildlife refuges, and BLM lands. Columbia University Press.

[cobi14331-bib-0064] Lynch, A. J. , Thompson, L. M. , Beever, E. A. , Cole, D. N. , Engman, A. C. , Hawkins Hoffman, C. , Jackson, S. T. , Krabbenhoft, T. J. , Lawrence, D. J. , Limpinsel, D. , Magill, R. T. , & Wilkening, J. L. (2021). Managing for RADical ecosystem change: Applying the Resist‐Accept‐Direct (RAD) framework. Frontiers in Ecology and the Environment, 19(8), 461–469.

[cobi14331-bib-0065] Lynch, A. J. , Thompson, L. M. , Morton, J. M. , Beever, E. A. , Clifford, M. , Limpinsel, D. , Magill, R. T. , Magness, D. R. , Melvin, T. A. , Newman, R. A. , Porath, M. T. , & Wilkening, J. L. (2022). RAD adaptive management for transforming ecosystems. Bioscience, 72(1), 45–56.

[cobi14331-bib-0066] Madin, E. M. , Darling, E. S. , & Hardt, M. J. (2019). Emerging technologies and coral reef conservation: Opportunities, challenges, and moving forward. Frontiers in Marine Science, 6, Article 727.

[cobi14331-bib-0067] Magness, D. R. , Hoang, L. , Belote, R. T. , Brennan, J. , Carr, W. , Stuart Chapin III, F. , Clifford, K. , Morrison, W. , Morton, J. M. , & Sofaer, H. R. (2022). Management foundations for navigating ecological transformation by resisting, accepting, or directing social–ecological change. Bioscience, 72(1), 30–44.

[cobi14331-bib-0068] Mahoney, J. , & Thelen, K. (Eds.). (2009). Explaining institutional change: Ambiguity, agency, and power. Cambridge University Press.

[cobi14331-bib-0069] Maitlis, S. , & Christianson, M. (2014). Sensemaking in organizations: Taking stock and moving forward. Academy of Management Annals, 8(1), 57–125.

[cobi14331-bib-0070] Martin, J. V. (2021). Between Scylla and Charybdis: Environmental governance and illegibility in the American West. Geoforum, 123, 194–204.

[cobi14331-bib-0071] Micelotta, E. , Lounsbury, M. , & Greenwood, R. (2017). Pathways of institutional change: An integrative review and research agenda. Journal of Management, 43(6), 1885–1910.

[cobi14331-bib-0072] Millar, C. I. , Stephenson, N. L. , & Stephens, S. L. (2007). Climate change and forests of the future: Managing in the face of uncertainty. Ecological Applications, 17(8), 2145–2151. 18213958 10.1890/06-1715.1

[cobi14331-bib-0073] Miller, B. W. , Schuurman, G. W. , Symstad, A. J. , Runyon, A. N. , & Robb, B. C. (2022). Conservation under uncertainty: Innovations in participatory climate change scenario planning from US national parks. Conservation Science and Practice, 4(3), Article e12633.

[cobi14331-bib-0074] Miller‐Rushing, A. , & Henkel, B. (2022). Climate‐smart restoration in Great Meadow, Acadia National Park. Wetland News, 32(5), 1–5.

[cobi14331-bib-0075] Milly, P. C. , Betancourt, J. , Falkenmark, M. , Hirsch, R. M. , Kundzewicz, Z. W. , Lettenmaier, D. P. , & Stouffer, R. J. (2008). Stationarity is dead: Whither water management? Science, 319(5863), 573–574. 18239110 10.1126/science.1151915

[cobi14331-bib-0076] Minato, W. , Curtis, A. , & Allan, C. (2010). Social norms and natural resource management in a changing rural community. Journal of Environmental Policy & Planning, 12(4), 381–403.

[cobi14331-bib-0077] Nadeau, C. P. , Hughes, A. R. , Schneider, E. G. , Colarusso, P. , Fisichelli, N. A. , & Miller‐Rushing, A. J. (2024). Incorporating experiments into management to facilitate rapid learning about climate change adaptation. Biological Conservation, 289I, Article 110374.

[cobi14331-bib-0078] National Environmental Policy Act, Public Law 91–190, 43 U.S. Code § 1638 et seq. (1970).

[cobi14331-bib-0079] National Forest Management Act (NFMA), Public Law 94–588, 16 U.S. Code § 1600 et seq (1976).

[cobi14331-bib-0080] National Park Service (NPS) . (2021a). Planning for a changing climate: Climate‐smart planning and management in the National Park Service . https://irma.nps.gov/DataStore/Reference/Profile/2279647

[cobi14331-bib-0081] National Park Service (NPS) . (2021b). Director's Order #2: Park Planning . https://www.nps.gov/subjects/policy/upload/DO_2_1‐11‐2021.pdf

[cobi14331-bib-0082] National Park Service Organic Act (An Act to establish a National Park Service, and for other purposes), Public Law 64–235, 54 U.S. Code § §1000 et seq. (1916).

[cobi14331-bib-0083] New Zealand Ministry for Culture and Heritage . (2017). The Treaty in brief . https://nzhistory.govt.nz/politics/treaty/the‐treaty‐in‐brief

[cobi14331-bib-0084] Nyborg, K. , Anderies, J. M. , Dannenberg, A. , Lindahl, T. , Schill, C. , Schlüter, M. , Adger, W. N. , Arrow, K. J. , Barrett, S. , Carpenter, S. , & Chapin III, F. S. (2016). Social norms as solutions. Science, 354(6308), 42–43. 27846488 10.1126/science.aaf8317

[cobi14331-bib-0085] Oakes, L. E. , Ardoin, N. M. , & Lambin, E. F. (2016). “I know, therefore I adapt?” Complexities of individual adaptation to climate‐induced forest dieback in Alaska. Ecology and Society, 21(2), Article 40.

[cobi14331-bib-0086] Page, R. , & Dilling, L. (2020). How experiences of climate extremes motivate adaptation among water managers. Climatic Change, 161, 499–516.

[cobi14331-bib-0087] Pascual, U. , Balvanera, P. , & Anderson, C. B. , Chaplin‐Kramer, R. , Christie, M. , González‐Jiménez, D. , Martin, A. , Raymond, C. M. , Termansen, M. , Vatn, A. , Athayde, S. , Baptiste, B. , Barton, D. N. , Jacobs, S. , Kelemen, E. , Kumar, R. , Lazos, E. , Mwampamba, T. H. , Nakangu, B. , … Zent, E. (2023). Diverse values of nature for sustainability. Nature, 620, 813–823. 37558877 10.1038/s41586-023-06406-9 PMC10447232

[cobi14331-bib-0088] Paxton, E. H. , Laut, M. , Enomoto, S. , & Bogardus, M. (2022). Hawaiian Forest bird conservation strategies for minimizing the risk of extinction: Biological and biocultural considerations (Technical Report HCSU‐103). Hawai'i Cooperative Studies Unit. https://dspace.lib.hawaii.edu/handle/10790/5386

[cobi14331-bib-0089] Persha, L. , Agrawal, A. , & Chhatre, A. (2011). Social and ecological synergy: Local rulemaking, forest livelihoods, and biodiversity conservation. Science, 331(6024), 1606–1608. 21436453 10.1126/science.1199343

[cobi14331-bib-0090] Persha, L. , Fischer, H. , Chhatre, A. , Agrawal, A. , & Benson, C. (2010). Biodiversity conservation and livelihoods in human‐dominated landscapes: Forest commons in South Asia. Biological Conservation, 143(12), 2918–2925.

[cobi14331-bib-0091] Pfaeffle, T. , Moore, M. A. , Cravens, A. E. , McEvoy, J. , & Bamzai‐Dodson, A. (2022). Murky waters: Divergent ways scientists, practitioners, and landowners evaluate beaver mimicry. Ecology and Society, 27(1), Article 41.

[cobi14331-bib-0092] Poland, M. , Hurwitz, S. , & McCleskey, R. B. (2022). How might the devastating June 2022 floods in and around Yellowstone National Park influence seismic and hydrothermal activity? U.S. Geological Survey. https://www.usgs.gov/observatories/yvo/news/how‐might‐devastating‐june‐2022‐floods‐and‐around‐yellowstone‐national‐park

[cobi14331-bib-0093] Pulver, S. , Ulibarri, N. , Sobocinski, K. L. , Alexander, S. M. , Johnson, M. L. , McCord, P. F. , & Dell'Angelo, J. (2018). Frontiers in socio‐environmental research. Ecology and Society, 23(3), Article 23.

[cobi14331-bib-0094] Radeloff, V. C. , Mockrin, M. H. , Helmers, D. , Carlson, A. , Hawbaker, T. J. , Martinuzzi, S. , Schug, F. , Alexandre, P. M. , Kramer, H. A. , & Pidgeon, A. M. (2023). Rising wildfire risk to houses in the United States, especially in grasslands and shrublands. Science, 382(6671), 702–707. 37943916 10.1126/science.ade9223

[cobi14331-bib-0095] Sayre, N. F. (2006). Ranching, endangered species, and urbanization in the Southwest: Species of capital. University of Arizona Press.

[cobi14331-bib-0096] Schoennagel, T. , Balch, J. K. , Brenkert‐Smith, H. , Dennison, P. E. , Harvey, B. J. , Krawchuk, M. A. , Mietkiewicz, N. , Morgan, P. , Moritz, M. A. , Rasker, R. , Turner, M. G. , & Whitlock, C. (2017). Adapt to more wildfire in western North American forests as climate changes. Proceedings of the National Academy of Sciences of the United States of America, 114(18), 4582–4590. 28416662 10.1073/pnas.1617464114 PMC5422781

[cobi14331-bib-0097] Schuurman, G. W. , Cole, D. N. , Cravens, A. E. , Covington, S. , Crausbay, S. D. , Hoffman, C. H. , Lawrence, D. J. , Magness, D. R. , Morton, J. M. , Nelson, E. A. , & O'Malley, R. (2022). Navigating ecological transformation: Resist–accept–direct as a path to a new resource management paradigm. Bioscience, 72(1), 16–29.

[cobi14331-bib-0098] Schuurman, G. W. , Hoffman, C. H. , Cole, D. N. , Lawrence, D. J. , Morton, J. M. , Magness, D. R. , Cravens, A. E. , Covington, S. , O'Malley, R. , & Fisichelli, N. A. (2020). Resist‐accept‐direct (RAD)—A framework for the 21st‐century natural resource manager (No. 2020/2213). National Park Service.

[cobi14331-bib-0099] Schuurman, G. W. , Hoving, C. L. , Hess, A. N. , Bristow, L. V. , Delphey, P. J. , Hellmann, J. J. , Keough, H. L. , Knutson, R. L. , & Kellner, A. (2023). Blue snowflakes in a warming world: Karner blue butterfly climate change vulnerability synthesis and best practices for adaptation (Natural Resource Report NPS/NRSS/CCRP/NRR—2023/2602). National Park Service. 10.36967/2301333

[cobi14331-bib-0100] Scott, T. (2023). Preservation or ‘Playing God?’ Flathead Beacon. https://flatheadbeacon.com/2023/06/21/glacier‐national‐park‐scientists‐seek‐to‐preserve‐native‐trout/

[cobi14331-bib-0129] Senese, A. , Azzoni, R. S. , Maragno, D. , D'Agata, C. , Fugazza, D. , Mosconi, B. , Trenti, A. , Meraldi, E. , Smiraglia, C. , & Diolaiuti, G. (2020). The non‐woven geotextiles as strategies for mitigating the impacts of climate change on glaciers. Cold Regions Science and Technology, 173, 103007.

[cobi14331-bib-0101] Sjöberg, L. (2000). Perceived risk and tampering with nature. Journal of Risk Research, 3(4), 353–367.

[cobi14331-bib-0102] Smith, S. L. , Cook, S. , Golden, A. , Iwane, M. A. , Kleiber, D. , Leong, K. M. , Mastitski, A. , Richmond, L. , Szymkowiak, M. , & Wise, S. (2022). Review of adaptations of US Commercial Fisheries in response to the COVID‐19 pandemic using the Resist‐Accept‐Direct (RAD) framework. Fisheries Management and Ecology, 29(4), 439–455. 35942481 10.1111/fme.12567 PMC9348349

[cobi14331-bib-0103] Steffens, R. (2023). Fire funding up nearly 25 percent in President's 2024 budget. Wildfire Today. https://wildfiretoday.com/2023/03/14/fire‐funding‐up‐nearly‐25‐percent‐in‐presidents‐2024‐budget/

[cobi14331-bib-0104] Stephenson, N. L. , & Millar, C. I. (2012). Climate change: Wilderness's greatest challenge. Park Science, 28(3), 34–38.

[cobi14331-bib-0105] Stirling, A. (2010). Keep it complex. Nature, 468, 1029–1031. 21179144 10.1038/4681029a

[cobi14331-bib-0106] St‐Laurent, G. P. , Hagerman, S. , & Kozak, R. (2018). What risks matter? Public views about assisted migration and other climate‐adaptive reforestation strategies. Climatic Change, 151(3‐4), 573–587.

[cobi14331-bib-0107] Swette, B. , & Lambin, E. F. (2021). Institutional changes drive land use transitions on rangelands: The case of grazing on public lands in the American West. Global Environmental Change, 66, Article 102220.

[cobi14331-bib-0108] Thaler, T. , Hanger‐Kopp, S. , Schinko, T. , & Nordbeck, R. (2023). Addressing path dependencies in decision‐making processes for operationalizing compound climate‐risk management. iScience, 26(7), Article 107073. 37416461 10.1016/j.isci.2023.107073 PMC10320201

[cobi14331-bib-0109] The Wilderness Act, Public Law 88–577, 16 U.S.C. 1131–1136 (1964).

[cobi14331-bib-0110] Thompson, L. M. , Lynch, A. J. , Beever, E. A. , Engman, A. C. , Falke, J. A. , Jackson, S. T. , Krabbenhoft, T. J. , Lawrence, D. J. , Limpinsel, D. , Magill, R. T. , Melvin, T. A. , & Wilkening, J. L. (2021). Responding to ecosystem transformation: Resist, accept, or direct? Fisheries, 46(1), 8–21.

[cobi14331-bib-0111] Ulibarri, N. , Cain, B. E. , & Ajami, N. K. (2017). A framework for building efficient environmental permitting processes. Sustainability, 9(2), Article 180.

[cobi14331-bib-0112] Ulibarri, N. , Escobedo Garcia, N. , Nelson, R. L. , Cravens, A. E. , & McCarty, R. J. (2021). Assessing the feasibility of managed aquifer recharge in California. Water Resources Research, 57(3), Article e2020WR029292.

[cobi14331-bib-0113] Ulibarri, N. , Goodrich, K. A. , Wagle, P. , Brand, M. , Matthew, R. , Stein, E. D. , & Sanders, B. F. (2020). Barriers and opportunities for beneficial reuse of sediment to support coastal resilience. Ocean & Coastal Management, 195, Article 105287.

[cobi14331-bib-0114] United States Department of the Interior (DOI) . (2021). Climate action plan . https://doi.gov/sites/doi.gov/files/department‐of‐interior‐climate‐action‐plan‐final‐signed‐508‐9.14.21.pdf

[cobi14331-bib-0115] United States Fish and Wildlife Service (USFWS) . (2022). Resist‐accept‐direct webinar series . U.S. Fish and Wildlife Service. https://www.fws.gov/training/webinar/resist‐accept‐direct‐framework

[cobi14331-bib-0116] United States Fish and Wildlife Service (USFWS) . (2023). Endangered and threatened wildlife and plants; designation of experimental populations. Federal Register, 88, 42642–42652. https://www.federalregister.gov/documents/2023/07/03/2023‐13672/endangered‐and‐threatened‐wildlife‐and‐plants‐designation‐of‐experimental‐populations

[cobi14331-bib-0117] United States Fish and Wildlife Service (USFWS) . (2024). A decision support framework for conservation introductions: U.S. Fish and Wildlife Service, Pacific Region. Pacific Region Conservation Introductions Working Group, U.S. Fish and Wildlife Service. 10.5281/zenodo.10456792

[cobi14331-bib-0118] United States Forest Service (USFS) . (2022). Confronting the wildfire crisis: A strategy for protecting communities and improving resilience in America's forests (FS‐1187a). https://www.fs.usda.gov/sites/default/files/fs_media/fs_document/Confronting‐the‐Wildfire‐Crisis.pdf

[cobi14331-bib-0119] U.S. Senate . (2023). United States Senate Bill 2272 . https://www.congress.gov/bill/118th‐congress/senate‐bill/2272

[cobi14331-bib-0120] Vogler, D. , Macey, S. , & Sigouin, A. (2017). Stakeholder analysis in environmental and conservation planning. Lessons in Conservation, 7, 5–16.

[cobi14331-bib-0121] Weick, K. E. (1995). Sensemaking in organizations (Vol. 3). Sage.

[cobi14331-bib-0122] Weirich, P. (2001). Decision space: Multidimensional utility analysis. Cambridge University Press.

[cobi14331-bib-0123] West, J. M. , Julius, S. H. , Kareiva, P. , Enquist, C. , Lawler, J. J. , Petersen, B. , Johnson, A. E. , & Shaw, M. R. (2009). US natural resources and climate change: Concepts and approaches for management adaptation. Environmental Management, 44, 1001–1021. 19636606 10.1007/s00267-009-9345-1 PMC2791483

[cobi14331-bib-0124] Willcock, S. , Cooper, G. S. , Addy, J. , & Dearing, J. A. (2023). Earlier collapse of Anthropocene ecosystems driven by multiple faster and noisier drivers. Nature Sustainability, 6, 1331–1342.

[cobi14331-bib-0125] Williams, J. W. , Ordonez, A. , & Svenning, J. C. (2021). A unifying framework for studying and managing climate‐driven rates of ecological change. Nature Ecology & Evolution, 5(1), 17–26. 33288870 10.1038/s41559-020-01344-5

[cobi14331-bib-0126] Wilson, M. A. (1997). The wolf in Yellowstone: Science, symbol, or politics? Deconstructing the conflict between environmentalism and wise use. Society & Natural Resources, 10(5), 453–468.

[cobi14331-bib-0127] Wilson, P. I. , Paveglio, T. , & Becker, D. (2018). The politically possible and wildland fire research. Fire, 1(1), Article 12.

